# Sweet Taste Signaling: The Core Pathways and Regulatory Mechanisms

**DOI:** 10.3390/ijms23158225

**Published:** 2022-07-26

**Authors:** Sunil Kumar Sukumaran, Salin Raj Palayyan

**Affiliations:** Nutrition and Health Sciences Department, University of Nebraska-Lincoln, Lincoln, NE 68583, USA; salin.rajp@unl.edu

**Keywords:** sweet taste receptor, gustation, G-protein-coupled receptor, noncaloric sweeteners

## Abstract

Sweet taste, a proxy for sugar-derived calories, is an important driver of food intake, and animals have evolved robust molecular and cellular machinery for sweet taste signaling. The overconsumption of sugar-derived calories is a major driver of obesity and other metabolic diseases. A fine-grained appreciation of the dynamic regulation of sweet taste signaling mechanisms will be required for designing novel noncaloric sweeteners with better hedonic and metabolic profiles and improved consumer acceptance. Sweet taste receptor cells express at least two signaling pathways, one mediated by a heterodimeric G-protein coupled receptor encoded by taste 1 receptor members 2 and 3 (*TAS1R2 + TAS1R3*) genes and another by glucose transporters and the ATP-gated potassium (K_ATP_) channel. Despite these important discoveries, we do not fully understand the mechanisms regulating sweet taste signaling. We will introduce the core components of the above sweet taste signaling pathways and the rationale for having multiple pathways for detecting sweet tastants. We will then highlight the roles of key regulators of the sweet taste signaling pathways, including downstream signal transduction pathway components expressed in sweet taste receptor cells and hormones and other signaling molecules such as leptin and endocannabinoids.

## 1. Introduction

It is thought that the taste system evolved to rapidly evaluate whether a potential food is suitable for consumption. Generally, five primary taste qualities are thought to exist: sweet, bitter, salty, sour, and umami ([1,2,3] and references therein). Although this classification captures all perceptually unique taste qualities, it does not include important classes of macronutrients such as fats, and minerals such as iron and calcium. It is now well recognized that the latter set of nutrients and others such as water do activate the taste system, although whether these findings warrant considering them as core taste qualities is still debated [2,3,4]. Another important caveat is that what we call taste in everyday language is in fact a combination of taste proper (gustation), smell (olfaction), and trigeminal chemosensation.

Carbohydrates are one of the fundamental building blocks of life, besides constituting a readily metabolizable source of energy. Although carbohydrate-sensing mechanisms, tuned primarily to their mono and disaccharide forms, are found in all kingdoms of life, their incorporation into the taste system evolved independently in both invertebrates such as insects and crustaceans and vertebrates ([4,5] and references therein). Sweet taste is perhaps the taste quality with the highest appetitive valence, and the innate preference for sweet-tasting foods arises very early in mammalian development [3]. Sweet taste presents one of the most interesting cases of plant–animal coevolution [6,7]. Plants pack their fruits with carbohydrates and sugars to provide a source of energy for the germinating seed. Animals, on the other hand, seek out fruits, as they are a rich source of sugar-derived calories and other nutrients such as vitamins and minerals. Although the destruction of the seeds by animals could be detrimental to plants, they also represent an opportunity for their dispersal. Thus, plants adapted by making hard-shelled seeds housed inside fleshy fruits that have a better chance of surviving the animal digestive system. This arrangement serves both parties well, and has unforeseen consequences as well; e.g., fruit-eating monkeys are thought to be evolutionarily more successful because they help in seed dispersal, ensuring a more stable food supply during lean times. However, the strong preference for sugars has turned to our detriment because most humans now live in an environment with far greater access to energy-rich foods, which is very different from that of our ancestors. With the spread of industrialization and colonialization, sugar plantations sprung up in many tropical colonies over the past 200 years, making this once luxury item extremely cheap and accessible [8]. More recently, high-fructose corn syrup made from starch has become a popular sugar source, especially in countries that cultivate a large amount of corn such as the USA, where it accounts for up to one-third of the caloric sugars used in processed foods [9]. Sugars such as sucrose and fructose and even glucose are not essential nutrients, unlike some amino acids and fats. However, the food industry was quick to latch on to our sweet tooth, and most processed and ultra-processed foods have unhealthily high levels of added sugars (and fats and salt), while simultaneously being low in beneficial but bitter-tasting phytonutrients and dietary fiber. This profoundly unhealthy diet has fueled a worldwide increase in conditions such as obesity and diabetes, placing an immense strain on the public health system and the wider economy ([10,11] and references therein). Noncaloric sweeteners (NCSs), compounds that taste sweet but have no caloric value, were among the first synthetic food additives to be approved [12,13]. However, the health benefits of existing NCSs are doubtful, and their consumer acceptance has still not attained full potential due to their off taste. In addition, they have been implicated in metabolic dysregulation, likely due to their post-ingestive signaling and adverse effects on intestinal microbiota [14,15]. In addition, strategies such as progressively reducing the sugar content of foods can reduce the taste threshold but not the pleasantness of sugars, although the underlying molecular mechanism(s) is/are not known [16]. Thus, there is a pressing need to learn more about the mechanisms of sweet taste signaling that may ultimately guide the development of novel NCSs with better health benefits and consumer acceptance. This review will focus on the pathways known to mediate sweet taste signaling, their mechanisms of regulation, and their physiological consequences and discuss some open questions in the field.

## 2. Organization of the Taste System

The taste system is exquisitely organized to sense and transduce taste signals to the brain. In mammals such as mice and humans, most taste papillae are distributed on the surface of the tongue, and isolated clusters are found on the soft palate and larynx as well [1,3,4]. Among them, the fungiform papillae (FFP), located on the anterior tongue each house a single taste bud, and the circumvallate (CVP) and foliate papillae (FOP), located medially and laterally on the back of the tongue, respectively, each contain clusters of multiple taste buds. Each taste bud is made up of 50–100 taste cells subdivided into at least four subtypes based on morphology (types I–IV) and at least five based on the taste quality they transduce signals of [1,17,18]. Type I cells are the most numerous, but they are the least well studied among taste cells. They are functionally analogous to glia in the nervous system and may transduce amiloride-sensitive salty taste. The type II cells consist of function subtypes that express G-protein-coupled receptors (GPCRs) for sweet, umami, or bitter tastants and their downstream signaling components [1,19]. They do not form typical synapses with taste nerves, but they secrete the neurotransmitter adenosine triphosphate (ATP) that binds to the purinergic receptors P2X2 and P2X3 in adjacent taste nerves [20]. The type III cells are analogous to neurons in that they form true synapses with taste nerves and secrete classical neurotransmitters such as 5-hydroxy-tryptamine and gamma amino butyric acid. They also generate true action potentials and possess voltage-gated calcium channels that trigger neurotransmitter release upon stimulation [21,22,23,24,25]. They include the functional subtypes that express receptors for sour and/or the amiloride-insensitive salty taste qualities [26]. Mature taste cells have half-lives ranging from 3 to 24 days and are continually regenerated from stem cells located at the base of taste buds [27]. Type IV cells are thought to be post-mitotic precursors of mature taste cells at an intermediate stage of differentiation [17,28,29]. Taste buds in the FFP are innervated by the chorda tympani branch of the facial (seventh cranial) nerve, and those in the CVP and FOP are innervated by the glossopharyngeal (ninth cranial) nerve that, together, transmit taste signals to the gustatory cortex through a multistep neuronal relay [1,4].

## 3. Pathways Mediating Sweet Taste Signaling

### 3.1. Sweet Taste Stimuli Are Structurally Diverse

Although sugars such as sucrose and fructose are the most well-known class of sweet taste stimuli, a large set of structurally unrelated molecules can elicit sweet taste, most of which are NCSs [13,30,31]. These include both small molecules and proteins derived from fruits and/or leaves of several tropical plants and synthetic chemicals, most of which are several hundreds to thousands-fold sweeter than sucrose on a molar basis [13,31]. The ecological significance of plant-derived NCSs is not known. It has been suggested that they represent a case of molecular mimicry that allows plants to substitute sugars for metabolically less costly molecules [32]. However, it is possible that some of them, such as steviol glycosides, may have other functions, such as acting as precursors for plant hormones, osmolytes, or feeding deterrents, and their sweetness might in fact be accidental [33]. In addition to the above agonists, a few sweet taste antagonists such as gymnemic acids, gurmarin, and lactisole are also known [34,35]. The mystery of how such a diverse set of molecules can elicit sweet taste was only solved after the discovery of the molecular mechanisms of sweet taste signaling, as described below.

### 3.2. The G-Protein-Coupled Receptor Pathway for Sweet Taste Signaling

#### 3.2.1. The Primary Sweet Taste Receptor Is a Heterodimer of Taste 1 Receptor Members 2 and 3 (TAS1R2 + TAS1R3)

Rodents are the preferred model system for molecular studies of taste transduction, due to the rarity of taste cells in the tongue (~1% of all cells in the lingual epithelium) and the consequent difficulty obtaining them from humans. Until the 2000s, it was believed that sweet taste is transduced by multiple receptors. The discovery of the expression of the G-protein alpha subunit *Gnat3* (G-protein subunit alpha transducin 3, aka gustducin) and the subsequent demonstration that *Gnat3* knockout mice have diminished sweet taste responses strongly suggested that GPCRs mediate sweet taste signaling [36,37]. Genetic studies in mice using sucrose-preferring strains such as C57BL/6 showed strong association of this trait with the *Sac* locus in chromosome 4, which was later shown to encode the GPCR *Tas1r3* (taste 1 receptor member 3) [38,39,40]. Further molecular studies identified the *Tas1r2* subunit of the receptor belonging to the same gene family [38]. Heterologous expression studies confirmed that the sweet taste receptor (STR) is a heterodimer of the two subunits (TAS1R2 + TAS1R3) [38,41]. TAS1R2 by itself is incapable of signal transduction; however, a homodimer of TAS1R3 may transduce sweet taste signals from natural sugars such as high concentrations of sucrose [42]. TAS1Rs belong to the class C subfamily of GPCRs that also includes the metabotropic glutamate receptors (*mGlurs*), vomeronasal receptors type 2 (*V2rs*), and the calcium-sensing receptor [43]. Like other members of the family, T1R2 and T1R3 possess at their amino terminus a Venus flytrap domain (VFT) composed of two lobes separated by a large cleft, followed by a short cysteine-rich domain (CRD), a seven transmembrane (7TM) domain, and a short intracellular domain at its carboxy terminus [41,44]. The TAS1R family consists of one other member, TAS1R1 that heterodimerizes with TAS1R3 to form the umami taste receptor. Using systematic mutagenesis studies of heterologously expressed STR, it was shown that it is capable of binding to all known sweet tastants and inhibitors [41,45,46]. These studies also helped identify the locations of the binding sites for the ligands distributed among the VFT, CTD, and 7TM domains of the T1R2 and T1R3 subunits [41,44,45,46]. Collectively, these studies demonstrated how the STR acts as a receptor for all known classes of sweeteners. However, this does not preclude the existence of other pathways for sweet taste signaling, as described in Section 3.3.

#### 3.2.2. STR-Mediated Signal Transduction Pathways

**Figure 1:**
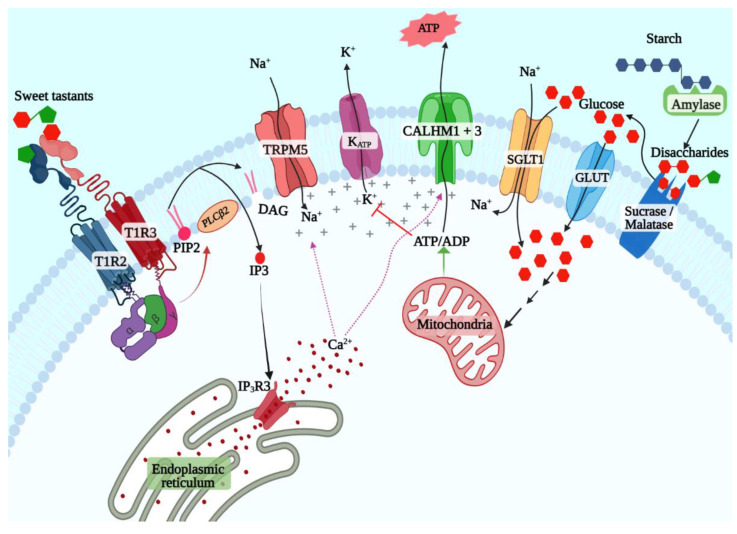
T1R2 + T1R3-dependent and -independent sweet taste signaling pathways. TAS1R2 + TAS1R3 is the primary sweet taste receptor that can bind to all known classes of sweet taste stimuli to activate the inositol 3-phosphate signaling pathway (left). This pathway triggers the release of calcium from the endoplasmic reticulum, which leads to opening of the monovalent cation-selective TRPM5 channel, which causes membrane depolarization by allowing the influx of sodium ions. Depolarization and elevated levels of calcium cause ATP release through the large pore channel formed by CALHM1 and CALHM3. The K_ATP_- and SGLT1 pathways, on the other hand, are selective for caloric sugars in the monosaccharide form (right). Complex starch is broken down into maltose by salivary amylase in the mouth. Maltose and other dietary disaccharides such as sucrose are broken down into monosaccharides by maltase and sucrase. Cotransport of sodium and glucose by SGLT1 induces membrane depolarization, while the K_ATP_ pathway requires catabolism of glucose to generate ATP through glycolysis, tricarboxylic acid cycle, and the electron transport chain, which then inhibits the K_ATP_ channel, triggering membrane depolarization.

Early studies from the 1970s onwards implicated cyclic adenosine (cAMP) as the key second messenger for sweet taste signaling [47]. The expression of adenyl cyclases and phosphodiesterases and sweetener-evoked cAMP generation in taste buds was demonstrated in taste cells from multiple species [48,49,50]. However, more recent studies have shown that the inositol 1,4,5-triphosphate (IP3) pathway is the primary pathway downstream of the STR (Figure 1) [1,51]. The cAMP pathway might play a regulatory role or be more relevant for signaling evoked by a subset of ligands such as caloric sugars. Ligand binding to the STR leads to the exchange of guanosine-5′-triphosphate (GTP) for GDP by the Gα subunit, leading to its dissociation from the Gβγ subunit [52]. The latter activates phospholipase C β2 (PLCβ2), which cleaves phosphatidylinositol 4,5-bisphosphate to diacyl glycerol and IP3 [51]. IP3 binds to its receptor (ITPR3) expressed on the membrane of the endoplasmic reticulum, inducing the release of Ca^2+^ [53]. The subsequent elevation of intracellular Ca^2+^ levels cause opening of the monovalent cation-selective channel transient receptor potential cation channel subfamily M member 5 (TRPM5), which leads to sodium influx and membrane depolarization [54,55]. Depolarization triggers the release of the neurotransmitter adenosine triphosphate (ATP) through a heterodimeric channel formed by the calcium homeostasis modulator 1 (CALHM1) and CALHM3 subunits, which activates the purinergic receptors in the taste nerves, leading to transmission of taste information to the brain [56,57].

#### 3.2.3. STR-Independent Sweet Taste Signaling Pathways

Numerous studies since the discovery of the STR have affirmed its primacy in sweet taste signaling. However, behavioral and taste nerve recording experiments showed that *Tas1r3* knockout mice retain responses to caloric sugars, while their responses to NCSs are almost completely abolished [58]. This was also observed in another study using *Tas1r2* and *Tas1r3* knockout mice, although *Tas1r2* + *Tas1r3* double-knockout mice did not show responses to sugars [59]. Similar results were also observed in knockout mice lacking *Gnat3* and *Trpm5* as well [37,60,61,62]. Since TAS1R2 by itself is incapable of responding to sweeteners, it raised the possibility that STR-independent caloric sugar-specific taste signaling pathways exist in taste cells [41]. Two such pathways have been well studied in other tissues: the ATP-sensitive potassium channel (K_ATP_)-dependent pathway first identified in pancreatic beta cells and the sodium–glucose cotransporter (SGLT) family mediated pathway first identified in the enteroendocrine cells in the small and large intestine. The latter is the simpler of the two; SGLT1 cotransports sodium ions and glucose (or galactose) from the intestinal lumen in a 2:1 ratio, leading to depolarization and action potential generation and secretion of the peptide hormones such as glucagon-like peptide-1 (GLP1) and GLP2 from enteroendocrine cells [63]. The K_ATP_ pathway depends on the generation of ATP from glucose transported into the beta cells. Several features of this pathway ensure that it serves a sensory role in the beta cells. Beta cells express the low-affinity facilitative glucose transporter GLUT2 and the low-affinity hexokinase glucokinase, both of which are active only at physiologically high glucose concentrations (above 5.5 mmol/L) that occur after a meal in nondiabetic individuals [64,65,66]. Phosphorylated glucose is metabolized through glycolysis and citric acid cycle, thus elevating the [ATP]/[ADP] ratio, leading to the closure of the K_ATP_ channel, depolarization of the cell membrane, and secretion of insulin. Since then, the K_ATP_ pathway was shown to be a potent glucose sensor in several other cell types, including the pancreatic alpha and delta cells, enteroendocrine cells, and glucose-sensing neurons in the hypothalamus as well, where it mediates a variety of responses such as gut hormone or neurotransmitter secretion depending on the cell type in question.

Several lines of evidence indicate that both these pathways are active in sweet taste cells [67,68,69,70,71]. A copious amount of amylase is secreted by salivary glands, enabling the production of maltose from starch in the oral cavity [72,73]. Similarly, the disaccharidases maltase-glucoamylase (MGAM, aka maltase) and sucrase-isomaltase (SI, aka sucrase), first identified in the brush border of enterocytes, are also expressed in taste cells and may generate glucose and fructose from maltose and sucrose in the vicinity of taste cells (Figure 1) [67]. Glucose transporters, including SGLT1 and several members of the GLUT family and the K_ATP_ channel, are coexpressed with *Tas1r3* in sweet taste cells (Figure 1) [69,71]. Behavioral and single-fiber taste nerve recording studies in mice showed that sweet taste responses are enhanced by additions of low concentration (10 mM) of sodium chloride, and this could be inhibited by the addition of phlorizin, an inhibitor of SGLT1 but not of GLUT family transporters [68]. These results were also confirmed in humans using psychophysical studies [74]. Similarly, K_ATP_-mediated currents were demonstrated in murine taste cells, and the taste nerve responses to caloric sugars in *Tas1r3* knockout mice were abolished by applying voglibose, an inhibitor of maltase and sucrase confirming the presence of the caloric sugar pathway in sweet taste signaling [67,69].

### 3.3. Physiological and Behavioral Significance of Sweet Taste Signaling through Multiple Pathways

The STR-independent and -dependent pathways have overlapping but distinct functional properties. As mentioned above, the STR is sensitive to both sugars and NCSs, while the SGLT1 and K_ATP_ pathways are tuned exclusively to caloric sugars. The sensitivity, kinetics of activation, and adaptation of these pathways are different. The STR has comparatively low sensitivity to caloric sugars such as sucrose and glucose (Km of 62 mM for sucrose, and above 100 mM for glucose) [75,76]. However, the Km of SGLT1 for glucose is 1.5 mM, and that of GLUT2 is >6 mM, enabling them to sense sugars at much lower concentrations than the STR [65]. Thus, it is possible that they are more relevant to sensing the low concentrations of monosaccharides generated from starch in the mouth during mastication [72]. The STR is subject to faster adaptation by endocytosis, as discussed below, while the membrane localization of SGLT and GLUT family members is increased in the plasma membrane (PM) upon glucose stimulation [77,78]. Interestingly, in the enterocytes, mRNA and protein levels and PM localization of SGLT1 are augmented by STR signaling in the neighboring enteroendocrine cells [79]. The STR pathway mediates the behavioral attraction to sugars, while the behavioral output of the K_ATP_ pathway is still under investigation. Sodium–glucose cotransport by SGLT1 may explain the enhancement of sweet taste by low concentrations of salt [69]. Interestingly, the K_ATP_ pathway was shown to mediate cephalic-phase insulin responses (CPIR) in mice [80,81]. In humans, oral carbohydrate, but not NCS, stimulation caused an increase in motor output in subjects undertaking fatigue-inducing exercise [82]. The above findings indicate that sugars and NCSs may activate at least partially nonoverlapping neural pathways and, consequently, may mediate different behavioral and physiological responses. The STR is also expressed in nutrient-sensing tissues throughout the body, such as the enteroendocrine cells, pancreas, and hypothalamus, where it is involved in regulation of incretin and insulin secretion and in regulating energy balance and food intake. These extraoral roles of the STR, coupled with its roles in taste and CPIR induction, might be the reason why sweet taste signaling is linked to metabolic conditions. Interestingly, high-carbohydrate or fat-fed *Tas1r3* knockout mice are resistant to development of obesity and hyperinsulinemia [83,84].

## 4. Regulation of Sweet Taste Signaling

Signaling by GPCRs is controlled by an array of GPCR-interacting proteins (GIPs) that act at various steps of the pathway, including at the receptor, G-proteins, or other downstream signaling components [85,86]. GIPs regulate various aspects of signaling such as anterograde transport and PM localization of GPCRs and G-proteins, their internalization, adaptation, and degradation and signaling kinetics [85,86]. GIPs of various classes described below are considered drug targets and may be targeted for developing novel classes of NCSs as well. One of the key mechanisms for regulating the K_ATP_ pathway is phosphorylation by protein kinase C and protein kinase A [87]. Protein kinases are activated by (GPCR mediated) IP3 and cAMP pathways, respectively, and may represent an opportunity for crosstalk between the two pathways. Interestingly, the expression of SGLT1 in enterocytes is regulated by STR signaling, although if this is the case in taste cells as well is not known [79].

### 4.1. Regulation of STR Anterograde Transport

**Figure 2:**
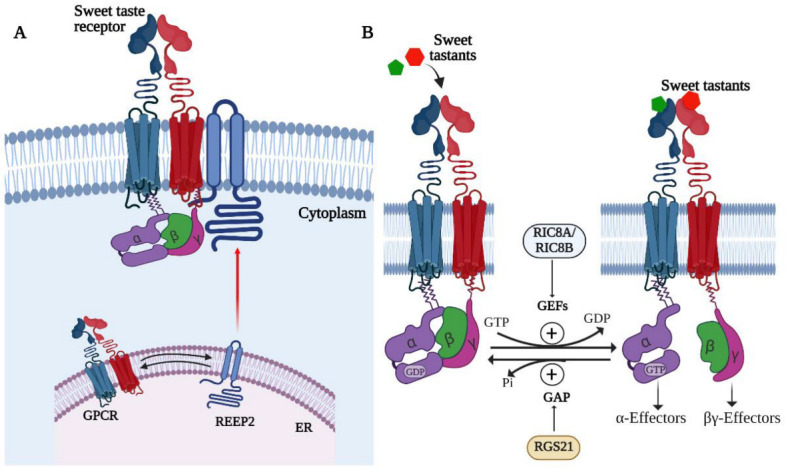
Accessory proteins regulating STR signaling. (**A**) REEP2 is a chaperone protein that noncovalently interacts with the STR and targets it to the plasma membrane. REEP2 may promote the localization of the STR to lipid rafts, which are thought to be specialized cholesterol and sphingolipid-rich microdomains in the plasma membrane where signaling complexes are assembled. (**B**) G-protein-interacting proteins regulate G-protein activity by modulating GTP binding or hydrolysis by the Gα subunit. Guanyl nucleotide exchange factors such as R1C8A and RIC8B may promote the exchange of GTP for GDP, thereby activating G-protein signaling, whereas GTPase-activating proteins such as RGS21 activate GTP hydrolysis to terminate G-protein signaling downstream of the STR. Part B adapted with permission from Siderowski and Willard [79].

Like all membrane proteins, GPCRs are synthesized, folded, and assembled at the endoplasmic reticulum (ER), from where they migrate to the ER–Golgi intermediate complex, the Golgi apparatus and the trans-Golgi network, and finally to the PM [88]. During this process, they may form dimers or multimers with their partner GPCRs or themselves and with GIPs and acquire post-translational modifications such as glycosylation [88]. In some cases, dimerization is essential for transport and PM localization, hence the partner GPCR can be considered a chaperone as well. Signal sequences at the C and N termini of GPCRs are required for interaction with GIPs to regulate their transport and membrane localization. The set of GIPs required for PM localization appears to be partially unique to each GPCR [89]. The failure to localize to the plasma membrane was an early hurdle to their functional expression in heterologous systems, which was only solved after identifying a suitable chaperone GIP. The major types of GIPs involved in anterograde transport include the homer proteins, small GTPases, receptor activity-modifying protein (RAMP), receptor-transporting protein (RTP), and receptor expression-enhancing protein (REEP) family members [88]. So far, the only GIP known to regulate STR localization to the PM is REEP2 [90]. REEP2 binds to the STR in taste cells and promotes its localization to lipid rafts-cholesterol and sphingolipid-rich PM microdomains that may represent signaling hotspots (Figure 2A). Consistent with this hypothesis, coexpression of REEP2 with STR in heterologous systems enhances sweet taste responses [90]. Incidentally, REEP2 enhances bitter taste responses when coexpressed with human bitter taste receptors as well. Similarly, an orthologue of the REEP family, REEP1 is essential for PM localization of odorant receptors [91].

### 4.2. STR Endocytosis and Adaptation

Perhaps the most well-studied mechanism that regulates GPCR signaling is mediated by arrestins. Arrestins are a small family of scaffolding proteins with pleiotropic effects on GPCR signaling [92,93,94,95]. The first identified role for arrestins was in desensitization of GPCRs. Upon activation and G-protein binding, GPCRs are phosphorylated by G-protein receptor kinases (GRKs); phosphorylated GPCRs are bound by arrestins, which sterically exclude further G-protein binding. Arrestins then recruit members of the endocytic machinery, including AP2 and clathrin to the complex. The receptor is then internalized into endosomes, which precludes further ligand binding and signaling. Endosomal GPCRs may be recycled to the PM or degraded. Interestingly, more recent studies have shown that GPCR-bound arrestins mediate signal transduction through the map kinase and the AKT pathways [94,95]. Thus, the role of arrestins in sweet taste signaling represents a promising avenue of research that has not been explored to date.

### 4.3. Promiscuous G-Protein Coupling Affect Signaling Pathways Downstream of the STR

The signaling pathway described in Section 3.2.2 is operational in all type II taste cells and mediates bitter and umami taste signaling as well. The α, β, and γ subunits of the G-protein are each encoded by gene families with several members, and it is not clear which combination of subunits is expressed in STR cells. GNAT3, the first identified Gα subunit in taste cells belonging to the G(t) subfamily of Gα proteins, can couple robustly with the STR in heterologous expression systems [38,96]. It is strongly coexpressed with the STR in the FFP but not in the CVP and FOP in mice [97]. Consistent with this observation, in *Gnat3* knockout mice, the sweet taste responses from the chorda tympani are almost fully abolished, but those from the glossopharyngeal nerve are less so [37]. Other G-proteins such as Gα14 (Gγ q family), Gαs, and Gαi are coexpressed with the STR in the CVP [98,99]. Thus, it is likely that the STR couples with multiple Gα subfamily members in native taste cells in various taste papillae. There is slightly better clarity on the identity of the Gβ and Gγ subunits, with GNB3 and GNG13 being the most well-known, although other γ and β subunits are also expressed in STR-expressing cells [52,100]. It is plausible that the cAMP or IP3 pathways may be differentially activated by sweet tastants based on the identity of the G-protein subunit genes the STR couples to, which could vary among taste papillae, and presumably among species. The identity of the subunit will also impact how G-protein signaling downstream of the STR is regulated, as described later. Among other downstream components, evidence exists that TRPM4 is coexpressed with TRPM5 in type II taste cells and could contribute to sweet, bitter, and umami taste signaling [101]. The pannexin 1 (PANX1) hemichannel is strongly expressed in type II taste cells and was thought to act as the ATP channel in taste cells [102]. Similarly, calcium- and integrin-binding protein 1 (CIB1) a regulator of ITPR3, was shown to bind the T1R2 and downregulate sweet taste signaling [103]. As described above, the identity of the components of the downstream signaling pathway in human taste cells is not well known and could differ from that in model organisms.

### 4.4. Roles of G-Protein Signaling Regulators

GPCR signaling by G-proteins is activated by exchange of GDP for GTP and terminated by hydrolysis of GTP to GDP bound to the Gα subunit. Indeed, the switch between GDP and GTP-bound forms of the subunit is perhaps the key step in regulation of GPCR signaling [85]. While the G-proteins themselves can catalyze both reactions at low levels, GIPs belonging to the guanine nucleotide exchange factors (GEFs) and GTPase-activating proteins (GAPs) respectively, accelerate these activities in vivo [85]. Members of both classes of proteins have been identified in type II taste cells. GEFs belong to the larger class of proteins called activators of G-protein signaling (AGS) that also include guanine nucleotide dissociation inhibitors and proteins that bind to the Gβγ complex. The GEFs RIC8A and RIC8B are coexpressed with ITPR3, a marker for type II taste cells [104]. RIC8A strongly interacts with GNAT3, Gαt2, and Gαi2 and was shown to amplify signaling downstream of human TAS2R16 in heterologous systems (Figure 2B) [104]. Whether it or other GEFs regulate sweet taste signaling is not known but remains an interesting possibility. RGS21 is expressed in type II taste cells, including those expressing the STR and bitter taste receptors [105]. RGS21 binds preferentially to the activated form of GNAT3 and other Gα subunits and enhances GTP hydrolysis, as expected (Figure 2B) [106]. RGS21 is also expressed in bitter taste receptor-expressing cells in the airway epithelium and was shown to tone down bitter taste receptor signaling in these cells [106]. Interestingly, global RGS21 deletion in mice caused reduced responses to bitter, sweet umami, and salty tastants [107,108]. This result is opposite of what is expected for a negative regulator and may reflect abnormalities in development and regeneration of the taste system or in expression of other taste signaling machinery components in the absence of this key signaling protein.

### 4.5. Interaction of G-Proteins with PDZ Domain-Containing Proteins May Regulate Their Microvillar Localization

G-proteins must be localized near the plasma membrane of the taste cell microvilli to participate in signaling. The taste cell microvilli are directly exposed to the epithelial surface, while the basolateral part of cells is sealed off from the epithelial surface by claudin protein-based tight junctions impervious to most solutes [109]. Signaling components downstream of the taste GPCRs such as TRPM5 and CALHM1 are localized to the basolateral surface of taste cells [110]. It appears that the tight compartmentalization of the signaling components in taste cells is necessary for efficient taste signaling, although how it is achieved is not well understood. PDZ domain-containing proteins are key regulators of transport and membrane localization of receptor proteins and signaling complex formation across all phyla [111]. Gγ13 interacts with multiple PDZ domain-containing proteins in taste cells, including PSD95, VELI-2 (LIN7B), SAP-97 (DLG1), GOPC, MPDZ, and ZO1, through its C-terminal tail region [112,113]. Some of these proteins are also involved in ER-PM transport, and a few have multiple PDZ domains and may interact with other signaling proteins and regulate signaling complex formation. Interestingly, in addition to GPCR signaling, G-proteins are known to regulate tight junction formation [85]. Thus, it is possible that their interaction with PDZ domain proteins has pleiotropic effects on taste signaling.

### 4.6. Hormonal Regulation of STR Signaling

**Figure 3:**
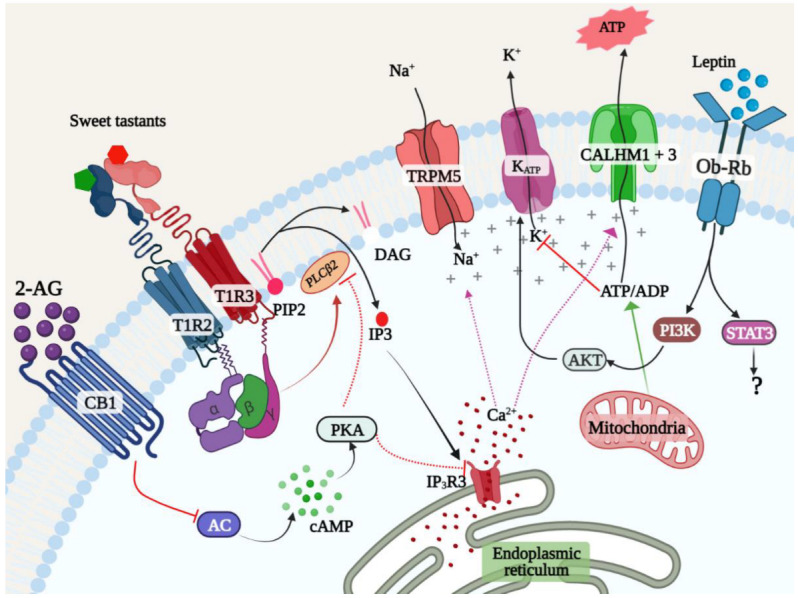
Leptin and endocannabinoids reciprocally regulate sweet taste signaling. The receptors for leptin and endocannabinoids are expressed in STR-expressing cells. Leptin signaling through the Ob-Rb receptor activates the PI3K–AKT signaling pathway in sweet taste cells to activate the K_ATP_ channel, thereby reducing the sensitivity of STR-expressing cells. Endocannabinoids signal through the CB1 receptor that is also expressed in these cells, to inhibit adenylyl cyclase, causing cAMP depletion and disinhibition of STR signaling. The action of these and other hormones regulates sweet taste signaling and behavioral responses to sweet tastants.

Hormones such as leptin and insulin and other signaling molecules such as endocannabinoids are key to translating the metabolic state to appropriate changes in physiology and behavior, including changes in appetite. Recent studies in mice have demonstrated that the anorexigenic (appetite suppressing) hormone leptin suppresses responses to both caloric sugars and NCSs [114,115,116]. Sweet taste cells coexpress the leptin receptor (LEPR, aka Ob-Rb) and the components of the downstream signal transduction machinery, including signal transducer and activator of transcription 3 (STAT3), which primarily mediates transcriptional responses and the phosphoinositide 3-kinase (PI3K)–AKT pathway [115,117]. It appears that the primary mechanism for suppression of sweet taste by leptin is by activation of the K_ATP_ channel through the PI3K–AKT pathway (Figure 3) [117]. Leptin induces the production of phosphatidylinositol (3,4,5)-trisphosphate (PIP3) and subsequent AKT phosphorylation in sweet taste cells [117]. In agreement with this observation, sulfonyl urea compounds that block the K_ATP_ channel eliminate leptin-mediated sweet taste suppression, while K_ATP_ activators augment it [116]. Interestingly, humans show a diurnal variation in sweet taste sensitivity, with the lowest recognition threshold (highest sensitivity) at 8.00 a.m. and the highest threshold at 8 pm, which is inversely correlated with circulating leptin levels [118].

Endocannabinoids such as 2-arachidonoyl glycerol (2AG) and anandamide ((N-arachidonoylethanolamine (AEA)) are orexigenic (appetite promoting) signaling molecules derived from fatty acids. Circulating levels of endocannabinoids are inversely correlated to that of leptin. They primarily act through the cannabinoid receptor 1 (CB1) to promote appetite. Like LEPR, CB1 is coexpressed with the STR in taste cells. Oral application of 2AG and AEA enhances taste nerve responses to both caloric sugars and NCSs in wild type, but not CB1 knockout mice [119,120]. The CB1 receptor is a GPCR that couples to G-proteins of the Gi/o pathway to inhibit adenylyl cyclase, lowering cAMP and thus inhibiting protein kinase A [121]. It is thought that inhibition of PKA disinhibits STR signaling (Figure 3). Thus, leptin and endocannabinoids have opposing effects on sweet taste signaling [120]. Interestingly, angiotensin II type 1 receptor (AT1) is also expressed in sweet taste cells and is known to heterodimerize with the CB1 receptor and synergize with sweet taste signaling [122]. In addition to the above hormones, the neuropeptides cholecystokinin (CCK) and Neuropeptide Y (NPY) and their receptors are coexpressed in taste cells, including sweet taste cells, and signals in an autocrine fashion to augment and suppress sweet taste signaling respectively [123,124]. CCK signaling in taste cells is thought to inhibit potassium channels, while NPY activates them [125]. Glucagon and glucagon-like peptides 1 and 2 on the other hand are secreted by STR-expressing cells in response to sweet tastants and stimulate taste nerves that express the cognate receptors [126]. GLP1R deficient mice have a profound deficiency in sweet taste sensitivity and heightened responses to umami tastants [127,128].

## 5. Conclusions

A clearer picture of the mechanisms regulating sweet taste signaling has emerged since the discovery of the sweet taste receptor and that of the caloric sugar-specific pathways, as well as the downstream signaling pathway components. NCSs currently in use have not proved very effective in reducing obesity and diabetes. Development of novel classes of NCSs may benefit from a thorough knowledge of the mechanisms regulating the core signaling pathway downstream of the sweet taste signaling pathways and the hormonal and other signaling pathways that modulate them.
